# Ruptured mitral valve aneurysm in a patient with immunodeficiency and chronic renal failure

**DOI:** 10.34172/jcvtr.026.33214

**Published:** 2026-03-30

**Authors:** Mustafa Kamil Yemis, Saadet Guven, Ozgur Can Usta, Ahmet Oz, Turgut Karabag

**Affiliations:** Department of Cardiology, Istanbul Education and Research Hospital, Saglik Bilimleri University, Istanbul, Turkey

**Keywords:** Mitral valve aneurysm, Rupture, Infective endocarditis, Immunodeficiency

## Abstract

Mitral valve aneurysm; defined as a bulge of the mitral valve leaflet toward the left atrium is a rare abnormality whose pathophysiology is poorly understood. The weakened aneurysmal wall may rupture and may cause severe mitral regurgitation and life-threatining conditions. In this case report we present a case of mitral anterior leaflet aneurysm rupture in a patient with immun deficiency and chronic renal failure.

## Introduction

 Mitral Valve Aneurysm (MVA) refers to a rare condition where the valve forms a saccular bulge towards the left atrium, swelling in systole and collapsing in diastole.^1,2^ While its occurrence is exceedingly rare ( < 0.3%), there are intriguing cases documented in the literature.^3^ Although MVA predominantly affects the anterior mitral valve, it can also originate from infection in the aortic valve,^4^ there have been sporadic reports of non-infectious causes contributing to MVA as well.^5,6^ We present a case of ruptured mitral valve aneurysm in a 56-year-old HIV-positive immunocompromised male patient with chronic renal failure.

## Case Presentation

 A 56-year-old male patient presented to the nephrology outpatient clinic with complaints of severe fatigue, cough, and shortness of breath. He reported increased fatigue recently, with shortness of breath even during minimal exertion, indicating New York Heart Association (NYHA) Class 3 symptoms. The patient had a history of undergoing dialysis three times a week for 12 years due to chronic kidney failure. Additionally, he was HIV-positive and had immune deficiency, was diabetic, and was receiving ongoing treatment for schizophrenia. Recently, he had been using a dialysis catheter as his left arm fistula was nonfunctional. His medication regimen included oral antidiabetics, antipsychotics, as well as dolutegravir and lamivudine for immune deficiency. Upon admission, his hemodynamics were stable, with normal vital signs (blood pressure: 146/70 mmHg, heart rate: 90 beats per minute, temperature: 36.7°C). Auscultation revealed a 3/6 systolic murmur in the mitral focus, while other system examinations were insignificant.

 In laboratory tests, the patient exhibited profound anemia with a hemoglobin level of 3.5 g/dL, alongside an elevated C-reactive protein (CRP) level of 189 mg/L. Procalcitonin levels were borderline high at 0.77 µg/L. The creatinine value was elevated at 3.7 mg/dL, with a glomerular filtration rate of 17 mL/min/m^2^. No significant abnormalities were observed on the electrocardiogram.

**Figure 1:**
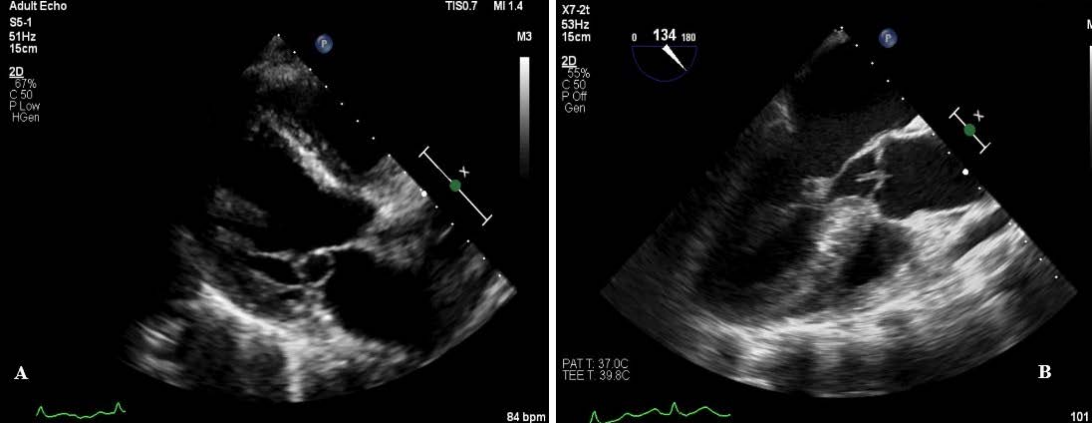
Figure showing a saccular structure protruding into left atrium (A) and a fibrillar structure extending from the aortic valve to the left ventricle (B) on transthoracic echocardiography

 With the current findings, the patient was admitted to the hospital for erythrocyte replacement and further examination and treatment. Transthoracic echocardiography was conducted to assess the murmurs detected on auscultation and to evaluate cardiac functions. The echocardiogram revealed normal left ventricular systolic function. Parasternal long-axis imaging showed dilation of the left atrium, left ventricular hypertrophy, and both aortic and mitral regurgitation with a saccular structure protruding into left atrium (Figure 1A, Supplementary File 1, Movie 1). In diastole, a fibrillar structure extending from the aortic valve to the left ventricle was observed (Figure 1B). In apical 4-chamber imaging, a structure entering and exiting the left atrium during systole was noted, potentially corresponding to a. Color Doppler examination showed central regurgitation as well as eccentric flow (Supplementary File 2, Movie 2). Following the initial findings, transesophageal echocardiography (TEE) was performed. During the TEE examination, a saccular aneurysmal formation originating from the anterior mitral valve and protruding into the left atrium during systole was observed. Additionally, a fibrillar structure originating centrally from the aortic valve was noted to enter and exit the left ventricle during diastole, resulting in moderate aortic regurgitation (Supplementary File 3, Movie 3). Color Doppler imaging revealed both central and eccentric regurgitation jets associated with the aneurysmal formation was seen. Also a regurgitation at the cleft of the aneurysm was clearly directed towards the left atrium. (Supplementary File 4, Movie 4).

 Based on the current findings, it was concluded that the structure observed in the aortic valve may be compatible with vegetation, which led to the development of an aneurysm in the anterior mitral valve, ultimately resulting in its rupture. Blood cultures were obtained from the patient, and antibiotic therapy with vancomycin (1g every 72 hours) and meropenem (500 mg every 8 hours) was initiated. The dialysis catheter insertion site was not infected. The nonfunctioning dialysis catheter was removed, a temporary dialysis catheter was placed, and the dialysis program continued. Transthoracic and transesophageal echocardiography revealed no vegetation on the dialysis catheter. The patient was also started on anticoagulation therapy because atrial fibrillation was detected on the electrocardiogram. Four units of erythrocyte replacement was applied. During follow-up, there was improvement in the patient’s infection parameters (CRP: 189 mg/L→14,42 mg/L, procalcitonin: 0.77 µg/L→0.43 µg/L), but there was no growth in blood cultures. Following consultation, mitral valve surgery was recommended. However, the patient and their family declined this option. Consequently, the patient continued antibiotic treatment for 1.5 months. A transthoracic echocardiogram performed at the end of this period revealed persistent issues with the mitral valve. However, it was observed that the structure in the aortic valve was disappeared. The patient declined the recommendation for transesophageal echocardiography. Written informed consent was obtained from the patient’s first-degree relatives fort he publication.

## Discussion

 Infective endocarditis (IE) is a severe and potentially life-threatening condition characterized by infection and inflammation of the endocardium, heart valves, cardiac devices, and catheters.^7^ It can readily infect and damage valvular tissues, occasionally leading to the formation of aneurysms, with a predilection for the anterior mitral valve.^8^ Although the exact mechanism remains unclear, it is believed that abscesses, granulation tissue, and scar formation, often resulting from local valve damage due to valvulitis, can manifest as sac-like structures under the influence of intraventricular pressure.^9^ Additionally, as observed in our case, aortic regurgitation may direct forces toward the anterior mitral valve, locally weakening the tissue and contributing to the formation of a sac-like structure.^10^ In some instances, spontaneous perforation may occur due to increased pressure, as seen in our case. Although the precise etiology of valve damage in our case is uncertain, it is plausible that the vegetative formation in the aortic valve, along with local spread and aortic regurgitation, may have propagated the infection to the anterior mitral valve. Furthermore, the patient’s immune deficiency likely facilitated the spread of the infection during this process.

 Imaging modalities, particularly transthoracic, transesophageal, and 3D echocardiography, play a crucial role in the diagnosis of mitral valve aneurysms, as in our case. Diagnostic features include the bulging of the aneurysmal sac into the left atrium during systole and its collapse in diastole, systolic flow into the saccular structure, diastolic reflux, and potential impingement of the aortic valve vegetation and resulting aortic regurgitation on the anterior mitral valve.^11^ In our case, besides eccentric mitral regurgitation, systolic flow from the ruptured aneurysmal sac into the left atrium was also observed. TEE serves as a crucial tool for excluding differential diagnoses such as mitral valve prolapse, myxomatous degeneration, and flail mitral, and it is considered more valuable than transthoracic echocardiography.^11^ In our case, the patient was diagnosed using both transthoracic echocardiography and TEE.

 The extent of mitral valve damage can vary depending on the location of valve involvement and the nature of the valve structure. In cases of posterior valve involvement, mitral valve damage may remain limited. However, large aneurysms involving the anterior mitral valve, particularly when accompanied by subvalvular apparatus involvement, can lead to significant valve displacement and serious damage.^12^ In such cases, surgical intervention is typically recommended. In our case, surgery was advised due to the perforation and advanced mitral regurgitation caused by damage to the anterior mitral valve. However, the patient declined surgery, and several factors, including their immunodeficiency status and psychological issues that could potentially impact the perioperative period, were taken into consideration. As a result, the decision was made to pursue antibiotic and medical management rather than surgical intervention.

## Conclusion

 Mitral valve aneurysms and subsequent rupture are critical and life-threatening complications associated with infective endocarditis. Particularly in individuals predisposed to infective endocarditis as in the patient we presented, prompt action should be taken upon suspicion raised by physical examination and laboratory findings. Imaging modalities, especially transthoracic echocardiography, play a pivotal role in diagnosis, enabling timely initiation of appropriate treatment measures.

## Competing Interests

 The authors declare no conflict of interest.

## Ethical Approval

 This study was approved by Research Ethics Committees of Saglik Bilimleri University Istanbul Education and Research Hospital

## Supplementary Files

Supplementary File 1, Movie 1. Saccular structure originating from mitral anterior leaflet and protruding into left atrium.

Supplementary File 2, Movie 2. Apical 5-chamber image showing central regurgitation as well as eccentric flow combined with aortic regurgitation.

Supplementary File 3, Movie 3. Transesophageal echocardiograpgy showing a saccular aneurysmal formation originating from the anterior mitral valve and protruding into the left atrium during systole and a fibrillar structure originating centrally from the aortic valve.

Supplementary File 4, Movie 4. Transesophageal echocardiograpgy showing both central and eccentric regurgitation jets associated with the aneurysmal formation. Also a regurgitation at the cleft of the aneurysm was clearly directed towards the left atrium was seen.
